# A Novel Study of Methane-Rich Gas Reforming to Syngas and Its Kinetics over Semicoke Catalyst

**DOI:** 10.1155/2014/707294

**Published:** 2014-05-15

**Authors:** Guojie Zhang, Aiting Su, Jiangwen Qu, Yannian Du

**Affiliations:** Key Laboratory of Coal Science and Technology, Taiyuan University of Technology, Ministry of Education and Shanxi Province, Taiyuan 030024, China

## Abstract

A small-size gasification unit is improved through process optimization to simulate industrial United Gas Improvement Company gasification. It finds that the reaction temperature has important impacts on semicoke catalyzed methane gas mixture. The addition of water vapor can enhance the catalytic activity of reforming, which is due to the fact that addition of water vapor not only removes carbon deposit produced in the reforming and gasification reaction processes, but also participates in gasification reaction with semicoke to generate some active oxygen-containing functional groups. The active oxygen-containing functional groups provide active sites for carbon dioxide reforming of methane, promoting the reforming reaction. It also finds that the addition of different proportions of methane-rich gas can yield synthesis gas with different H_2_/CO ratio. The kinetics study shows that the semicoke can reduce the activation energy of the reforming reaction and promote the occurrence of the reforming reaction. The kinetics model of methane reforming under the conditions of steam gasification over semicoke is as follows: $\overset{-}{k}=5.02\times 10^{3}\cdot p_{\text{C}{\text{H}}_{4}}^{0.71}\cdot p_{{\text{H}}_{2}}^{0.26}\cdot \text{exp}$(−74200/*RT*).

## 1. Introduction

China is one of the largest countries of coke production and consumption in the world. At the same time, a great amount of coke oven gas is produced. Part of the coke oven gas is used to maintain furnace temperature, but about 85 billion cubic meters are wasted without using [1, 2]. So the problem of how to use these resources on a clean and high-efficiency basis is the major concern of enterprises and researchers. Coke oven gas mainly consists of methane (23%–27%) and hydrogen (55%–60%), of which the latter can be separated via pressure swing adsorption, while methane-rich gas mixture can be utilized to produce synthesis gas. Methane-rich gas reforming and CO_2_ and H_2_O reforming are two main ways for methane conversion to synthesis gas.

However, the average bond energy of C–H is 4 × 10^5^ J/mol, and CH_3_–H bond dissociation energy also reaches 4.35 × 10^5^ J/mol. The methane molecules have strong stability and can be converted or decomposed only under high temperature or catalytic action. In recent years, researches on the catalyst for methane reforming are constantly emerging. In addition to precious metals and transition metals, carbon material is also found to possess a significant catalytic action to methane reforming and, therefore, has preferable anticarbon deposition property. Zhang et al. found that, under the catalytic action of carbon, the conversion rate of CH_4_ and CO_2_ was higher in the initial phase and then declined to the stable level [3]. The temperature rising is favorable to methane reforming. Li et al. found that, under the catalytic action of carbon, the specific surface area and ash content had great impacts on methane conversion and the specific surface area of coke would reduce more or less during the reaction process [4]. Sun et al. believed that the fixed carbon content of coke is the main catalytic component in methane conversion [5]. Through material balancing of carbon atom and hydrogen atom before and after reaction, Zhang et al. found that the action of semicoke in reforming reaction was similar to the effect of catalyst [6]. Those carbonaceous materials that have a high specific surface area show higher catalytic activity [7]. The research also found that the oxygen functional groups on the surface of carbonaceous materials are involved in the reforming and these groups along with pore structure on the surface are the major factors influencing the catalytic properties [8]. The research also found that the carbonaceous catalyst could reduce the activation energy of methane dehydrogenation [9]. We have found that coke modified by metal possessed a better catalytic activity to carbon dioxide reforming of methane in coke oven gas [10]. In addition, oxidative modification by H_2_O_2_ could improve the catalytic activity of coke [11]. Adjusting the partial pressure of methane can control the carbon-hydrogen ratio of the synthesis gas at the outlet [12]. The CH_4_/CO_2_ ratio of inlet gas can be regulated by adjusted H_2_/CO in the synthesis gas. According to the above-mentioned researches, this study used semicoke as the raw material for gasification and United Gas Improvement Company coal gasification as model. The small-size gasification unit is improved through process optimization to simulate industrial United Gas Improvement Company gasification. The methane-rich gas mixture is introduced in the up-blow stage to prepare synthesis gas. The influences of reaction temperature, semicoke particle size, and methane partial pressure on methane reforming to synthesis gas are studied. The dynamics of methane reforming to synthesis gas is observed under the semicoke condition.

## 2. Methods

### 2.1. Sample

Carbon material used in the research was provided by Town Star Industry Co., Ltd., in Linfen, Shanxi. The results of industrial analysis and elemental analysis are shown in Table 1. The composition of methane-rich gas mixture in the coking plant area is shown in Table 2, while the composition of methane-rich gas mixture for experiments is CH_4_ 54%, CO_2_ 8%, and H_2_ 3%.

### 2.2. Catalytic Activity Measurements

The reactions over the catalyst were carried out at normal pressure in a continuous-flow quartz reactor (i.d. 20 mm) packed with particle size of 2 mm, 18 g ~ 20 g catalyst, and provided with a platinum-rhodium thermocouple located in the center of the catalyst bed. The reactions were carried out at different temperatures, and relative concentrations of CH_4_, H_2_, and CO_2_ in the feed were measured at normal temperature and pressure. Before carrying out the reaction, the catalyst bed was heated in situ at 1100°C in a flow (50 cm^3 ^min^−1^) of moisture-free nitrogen, then heating was stopped, and methane-rich gas mixture and water vapor were introduced. The product gases were analyzed by GC-960 produced by Shanghai Haixin Co., Ltd. In the GC, a column of 2 m filled with carbon sieve was used at column temperature 90°C and TCD detector was used at detector temperature 70°C. The gasification room temperature was 120°C. The current flow was 40 mA, and the carrier gas is argon. And the output of product gas flow can be measured using soap liquid meter. The C, H, and O balance across the reactor was more than 97%. All experiments with larger errors in the material balances were rejected. Prior to analysis, the effluent was passed through a water-trap at 0°C in order to remove reaction water. The calculation equation of CH_4_ and CO_2_ conversion was followed:

(1) XCH4=FCH4in×YCH4in−FCH4out×YCH4outFCH4in×YCH4in×100%,XCO2=FCO2in×YCO2in−FCO2out×Y  CO2out  FCO2in×YCO2in×100%,

where *X* is the conversion of the CH_4_ or CO_2_, *F* is the gas flow rate of in and out, mL·min^−1^, *Y* is the different fraction volume percentage, superscript in is the inlet, and superscript out is the outlet.

After the catalyst had served the reaction for a specified period of time, the reaction feed was switched to inert nitrogen (high purity), followed by cooling in nitrogen flow of the reactor to room temperature at which the used catalyst was unloaded for various characterizations.

### 2.3. Catalyst Characterization

All samples were degassed at 573 K for 1 h before measurements to remove impurities of the catalyst surface. The specific surface area of the catalysts was determined by nitrogen adsorption-desorption measurement at −196°C in Tristar gas adsorption system.

Scanning electron microscopy (SEM) observations of the catalyst samples were performed using a JSM-4800 (Japan, JEOL Ltd.).

## 3. Results and Discussion

### 3.1. The Effect of Temperature on Methane Conversion

**Figure 1:**
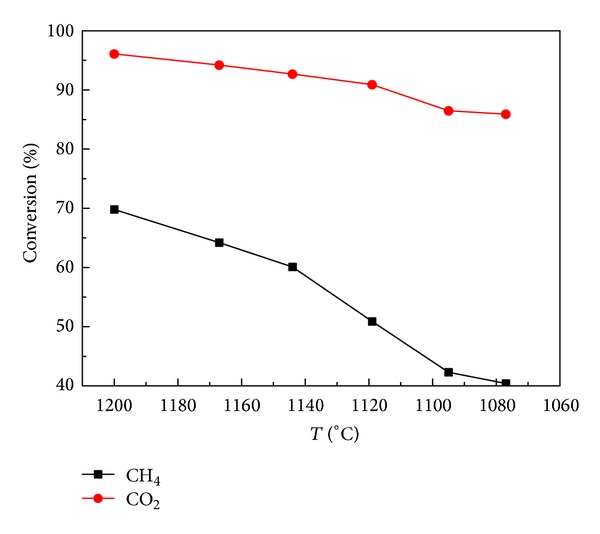
Effect of temperature on conversion of methane and carbon dioxide.

Under the conditions of atmospheric pressure, flow rate of 363 mL/min, and CO_2_/CH_4_ = 1, the influence of semicoke temperature on methane reforming is inspected, with the results shown in Figure 1. From the figure, it can be seen that the conversion rate of CO_2_ is obviously higher than that of methane. It is mainly because CO_2_ not only is involved in reforming reaction with methane under high temperature condition but also reacts with semicoke in gasification reaction. Moreover, Figure 3 also shows that, with the decline of temperature, the conversion rates of CH_4_ and CO_2_ decrease. The temperature has an important impact on the reforming reaction. The reason for such impact is that methane reforming reaction is a strong endothermic reaction. Therefore, the falling of reaction temperature is not beneficial for the activation of gas molecule. On the other hand, it also reduces the diffusion rate of the molecules and the molecular collision, which has an adverse impact on the proceeding of reforming reaction. As a result of this, the conversion rates of CH_4_ and CO_2_ decrease.

### 3.2. The Effect of Semicoke Particle Size on Methane Conversion

**Figure 2:**
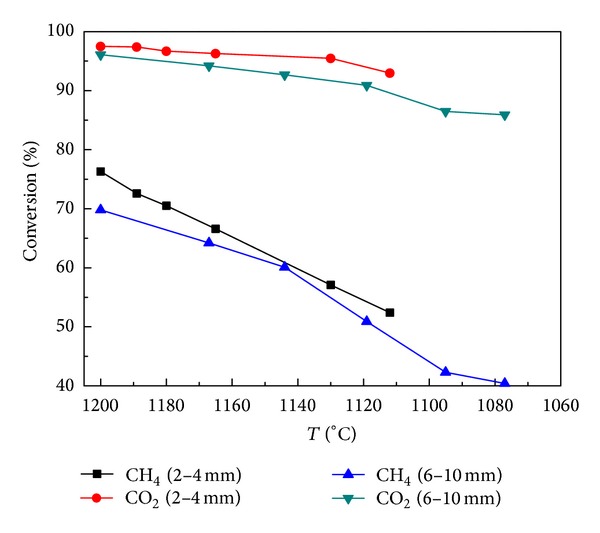
Effect of particle size on conversion of methane and carbon dioxide.

Under the conditions of atmospheric pressure, flow rate of 363 mL/min, and CO_2_/CH_4_ = 1, the influence of semicoke particle size on methane reforming is inspected, with the results shown in Figure 2. Figure 2 shows that the catalytic effect of particle size 2 mm is obviously better than that of 6 mm, and the main reason is the influence of diffusional resistance on the gases inside the pores. The smaller the particle size is, the lower the gas resistance is when passing through semicoke bed. The diffusion rate is higher, the effect of mass transfer is greater, and the effective reaction rate is faster (as the intrinsic rate is the same). For semicoke of the same mass, the one with a smaller particle size will have a larger specific surface area, which not only is favorable to the activation of molecular adsorption, but also results in more active sites per unit volume [13]. The reduction of catalytic activity is also less significant.

### 3.3. The Influence of Water Vapor on Methane Reforming Reaction

**Figure 3:**
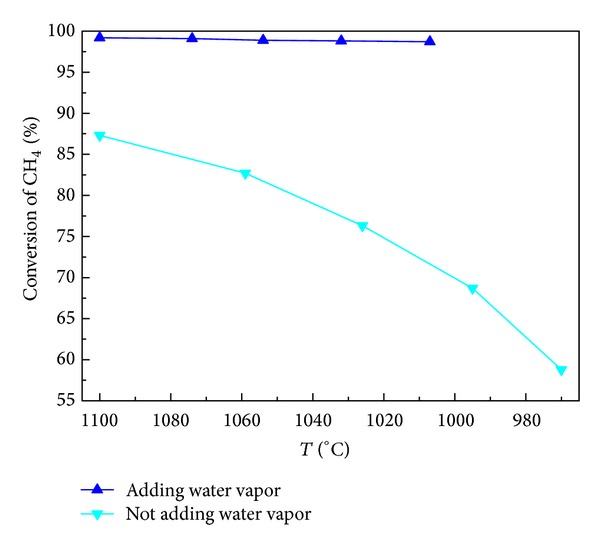
Effect of water vapor on conversion of methane.

**Figure 4:**
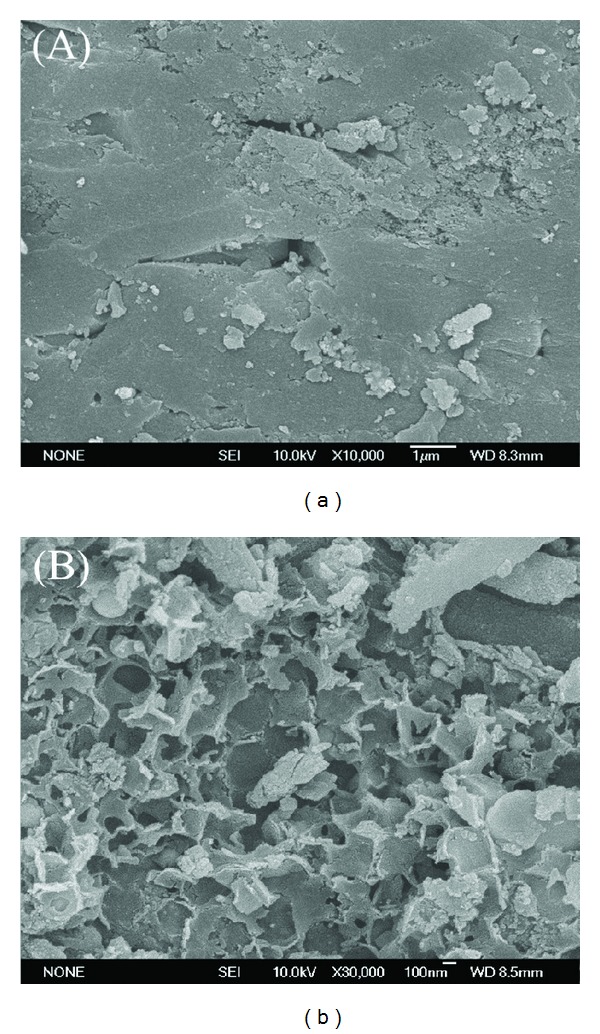
SEM of catalysts.

Under the conditions of atmospheric pressure and flow rate of 240 mL/min, CO_2_/CH_4_ = 1, and H_2_O/CH_4_ = 0.9, methane-rich gas mixture and water vapor are engaged in reforming reaction in the presence of semicoke. The methane conversion is illustrated in Figure 3. From the figure it can be seen that the addition of water vapor can clearly enhance the conversion rate of methane. In the condition without water vapor, the conversion rate of methane is 87% at 1100°C and it sharply declines with the falling of temperature. When water vapor is introduced at a certain flow rate, the conversion rate of methane could be maintained above 98%. There are four reasons for this phenomenon. The first reason is that carbon deposit produced by methane decomposition has certain activity in the initial phase and undergoes gasification reaction with water vapor under high temperature, reducing the generation of carbon deposit [14, 15]. Second, the gasification reaction takes place between water vapor and carbon bed under high temperature, which reduces the compactness of the carbon bed [16]. The images of semicoke surface before and after the reaction are shown in Figure 4. Surface area, pore volume and pore size of semi-coke are shown in Table 3. From Figure 4 and Table 3, it can be seen that semicoke surface changes greatly before and after the reaction. Before the reaction, the surface is regular and even, while the one after reaction is porous. Thus, the contact surface between gas and active sites of carbon material is enlarged, which is beneficial to the adsorption of methane and carbon dioxide molecules and the improvement of methane conversion rate. Thirdly, the gasification reaction between semicoke and water vapor results in the generation of many new active oxygen-containing functional groups, providing new active sites for methane conversion to enhance methane conversion rate [17]. Fourthly, the steam reforming of methane proceeds smoothly, and methane conversion rate increases.

### 3.4. The Influence of the Proportion of Methane on H_2_/CO Ration in the Syngas

**Figure 5:**
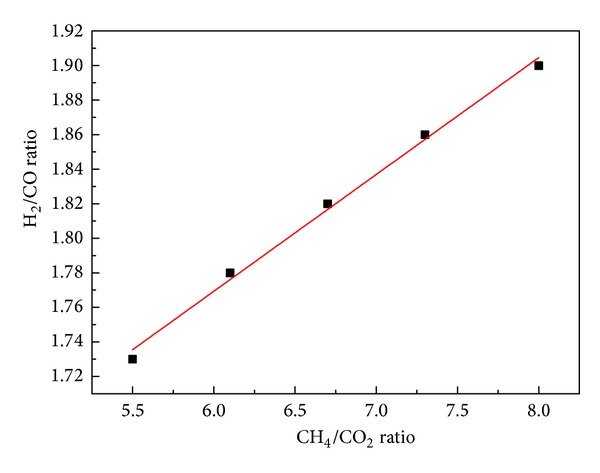
Effect of the proportion of CH_4_ on H_2_/CO ration in the syngas.

Under atmospheric pressure, flow rate of methane-rich gas mixture 343 mL/min, and temperature 1100°C, the influence of different CH_4_/CO_2_ ratios on H_2_/CO ratio is inspected, with the results shown in Figure 5. It can be seen that methane-rich gas mixture with different proportions of methane presents a good linear relation with H_2_/CO ratio. The H_2_/CO ratio is 1.7 when methane is added, while the H_2_/CO ratio is about 1.3 under the same condition when only water vapor is added. Therefore, the synthesis of different chemical products has diverse feed gas requirements in Fischer-Tropsch synthesis. That is, H_2_/CO ratio should range between 1 and 2. The addition of methane-rich gas mixture in the gasification process can regulate H_2_/CO ratio in the synthesis gas. This not only enables a full use of raw material, but also reduces water gas conversion and the emission of carbon dioxide, which will contribute to energy conservation and emission reduction.

## 4. Kinetics

### 4.1. Establishment of the Model

During the water vapor gasification of carbon, the methane-rich gas mixture is mainly involved in the following reactions:

(2) $$\begin{array}{c} {\text{CH}}_{4}+{\text{CO}}_{2}-2\text{CO}+2{\text{H}}_{2}\\ {\text{CH}}_{4}+{\text{H}}_{2}\text{O}-\text{CO}+3{\text{H}}_{2}\\ \text{CO}+{\text{H}}_{2}\text{O}-{\text{CO}}_{2}+2{\text{H}}_{2}\\ \text{C}+{\text{H}}_{2}\text{O}-\text{CO}+{\text{H}}_{2}\\ \text{C}+2{\text{H}}_{2}\text{O}-{\text{CO}}_{2}+{\text{H}}_{2}\\ \text{C}+{\text{CO}}_{2}-2\text{CO} \end{array}$$

Due to the multiplicity of reactions in this system, the following presumptions are made for the model to study methane conversion: methane reforming reaction is the main reaction and only the influences of temperature and initial concentration of reactants on conversion rate are considered, regardless of the intermediate products [18].

When water vapor is introduced, the methane consumption rate is expressed as the function of reaction rate and reaction time. Whether the maximum rate occurs or not is not considered. The reaction rate is expressed by

(3) $$\begin{array}{c} f=1-\exp\left(-a\theta^{b}\right). \end{array}$$

Reaction rate *K*_*f*_ is defined by

(4) $$\begin{array}{c} K_{f}=\frac{df}{d\theta }=k\left(1-f\right). \end{array}$$

The integration of formula (4) yields

(5) $$\begin{array}{c} f=1-\exp\left[-\overset{\theta }{\underset{0}{\int }}k\left(\theta \right)\text{d}\theta \right]. \end{array}$$

The comparison of formula (3) and formula (5) yields

(6) $$\begin{array}{c} k\left(\theta \right)=ab\theta^{b-1}. \end{array}$$

By establishing simultaneous equations with formula (3), we get

(7) $$\begin{array}{c} k\left(f\right)=a^{1/b}\cdot b{\left[-\ln\left(1-f\right)\right]}^{\left(b-1\right)/b}. \end{array}$$

By whole-process integration of formula (7), the following is obtained:

(8) $$\begin{array}{c} \bar{k}=\overset{1}{\underset{0}{\int }}k\left(f\right)df⟶\overset{0.99}{\underset{0.01}{\int }}k\left(f\right)df. \end{array}$$

The relations between average rate constant and partial pressure of gas component *P* and between temperature *T* and activation energy *E* are further assumed as in formula (9).

A logarithm is taken on both sides of the equation:

(9) $$\begin{array}{c} \bar{k}=k_{0}\cdot p_{{\text{CH}}_{4}}^{m}\cdot p_{{\text{H}}_{2}}^{n}\cdot \exp\left(-\frac{E}{RT}\right). \end{array}$$

ln⁡*k* − 1/*T* is plotted to get the activation energy of reaction. By establishing three simultaneous equations, the value of *k*_0_ is known.

### 4.2. Dynamic Results

**Figure 6:**
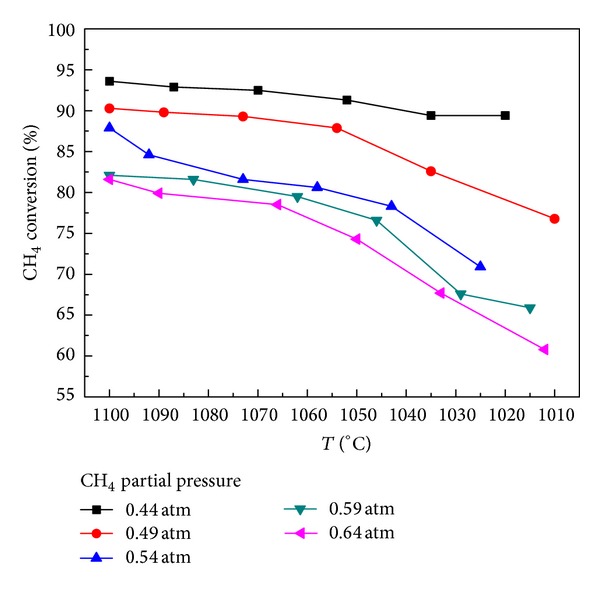
The effect of different proportions of methane on CH_4_ conversion.

Under the conditions of atmospheric pressure and inlet gas flow rate 343 mL/min, methane conversion is analyzed under different temperatures. At methane partial pressure of 0.44 atm, CO_2_ partial pressure of 0.08 atm, and hydrogen partial pressure of 0.28 atm, the methane conversions are shown in Figure 6, respectively. It can be seen that, under the same conditions, if methane partial pressure is larger, the conversion rate would be lower; with the falling of temperature, the conversion rate shows a downward trend. The values are introduced into formula (9) to get the relevant parameters, as shown in Table 4.

The apparent activation energy for methane consumption in this system is 74.2 kJ·mol^−1^, but Meng et al. calculated the value to be 94.5 kJ·mol^−1^ by theoretical analysis and simulation of reforming of methane-rich gas conversion to synthesis gas in the presence of semicoke [19]. It is clear that the addition of water vapor is favorable to methane conversion.

## 5. Conclusions

Based on United Gas Improvement Company coal gasification technology, a small-size gasification unit is improved through process optimization to simulate industrial United Gas Improvement Company gasification. The results show that the reaction temperature has important impacts on semicoke catalyzed methane gas mixture. The addition of water vapor can enhance the catalytic activity of reforming, which is due to the fact that addition of water vapor not only removes carbon deposit produced in the reforming and gasification reaction processes, but also participates in gasification reaction with semicoke to generate some active oxygen-containing functional groups. The active oxygen-containing functional groups provide active sites for carbon dioxide reforming of methane, promoting the reforming reaction. It also finds that the addition of different proportions of methane-rich gas can yield synthesis gas with different H_2_/CO ratio. The kinetics study shows that the semicoke can reduce the activation energy of the reforming reaction and promote the occurrence of the reforming reaction. The kinetics model of methane reforming under the conditions of steam gasification over semicoke is as follows:

(10) $$\begin{array}{c} \overset{-}{K}=5.02\times 10^{3}\cdot p_{{\text{CH}}_{4}}^{0.71}\cdot p_{{\text{H}}_{2}}^{0.26}\cdot \exp\left(-\frac{74200}{RT}\right). \end{array}$$

## Figures and Tables

**Table 1:** The proximate analysis and ultimate analysis of carbon catalyst.

Sample	Proximate analysis, wt, %, ad			Ultimate analysis, wt, %, daf				
	Moisture	Ash	Volatile matter	C	H	N	S	O (diff)
Semicoke	0.54	15.89	2.09	81.29	0.61	0.60	1.33	0.28

ad: air dried; daf: dry ash free; diff: difference.

**Table 2:** Composition of methane-rich gas.

Composition	O_2_	N_2_	CH_4_	CO	CO_2_	C_m_H_n_	H_2_
Content, %	0.91	5.63	53.88	19.31	8.05	6.63	5.59

**Table 3:** Surface area, pore volume, and pore size of semicoke.

Sample	Surface area/m^2^ g^−1^	Pore volume/cm^3^ g^−1^	Pore size/nm
Before reaction	15.192	0.009	8.735
After reaction	18.418	0.013	7.116

**Table 4:** Kinetic parameters.

*k0 *	*m *	*n *	*E*/Kj·mol^−1^
5.02 × 10^3^	0.71	0.26	74.2
